# Effects of the interaction of Notch and TLR4 pathways on inflammation and heart function in septic heart

**DOI:** 10.1515/biol-2022-0076

**Published:** 2022-07-13

**Authors:** Ziyang Liu, Wenli Li, Yang Cao, Xiaoxia Zhang, Kai Yang, Fukang Yin, Meng Yang, Peng Peng

**Affiliations:** Intensive Care Unit, Emergency Trauma Center, The First Affiliated Hospital of Xinjiang Medical University, Urumqi 830011, Xinjiang, China; Emergency Department of Internal Medicine, Emergency Trauma Center, The First Affiliated Hospital of Xinjiang Medical University, Urumqi 830011, Xinjiang, China

**Keywords:** sepsis, myocardial injury, TLR4, notch, NF-κB

## Abstract

We investigated the role of the interaction between the Notch and Toll-like receptor 4 (TLR4) pathways in septic myocardial injury. The sepsis model was induced in rats with lipopolysaccharide (LPS). Rats were divided into control, LPS, LPS + TAK242 ((6*R*)-6-[*N*-(2-chloro-4-fluorophenyl)sulfamoyl]cyclohex-1-ene-1-carboxylate) and LPS + DAPT (*N*-[*N*-(3,5-difluorophenacetyl)-l-alanyl]-*s*-phenylglycinetbutylester) groups. Heart function was evaluated with a Cardiac Doppler ultrasound. Myocardial morphological changes were detected by hematoxylin-eosin staining (H&E). Apoptosis was assessed by a TUNEL assay. The mRNA and protein levels were detected with real-time PCR, Western blot, and immunohistochemistry analysis. We found that heart function in the LPS + TAK242 group was significantly improved, but not in the LPS + DAPT group. LPS + TAK242 had a lower level of degeneration and necrosis of cardiomyocytes and inflammatory cell infiltration, as well as lower apoptosis and caspase-3 expression than the LPS group. Compared with the LPS group, the inflammatory cell infiltration was reduced in the LPS + DAPT group, while the degeneration and necrosis of cardiomyocytes were not obviously improved. Additionally, the expression levels of tumor necrosis factor-α and Interleukin-6, the protein contents of Notch intracellular domain and Hes1, and the P65 nuclear factor kappa-B (NF-κB) to P-P65 NF-κB ratio in LPS + TAK242 group and LPS + DAPT group were significantly lower than those in LPS group. Conclusively, the interaction between TLR4 and Notch signaling pathways enhances the inflammatory response in the septic heart by activating NF-κB. Blocking the TLR4 pathway with TAK242 can improve heart dysfunction and myocardial damage in sepsis, while blocking the Notch pathway with DAPT cannot effectively prevent heart dysfunction and myocardial damage in sepsis.

## 1 Introduction

Sepsis is defined as the life-threatening multiple organ dysfunction caused by the imbalance of the host response to infection [1], which has been recognized as the main cause of death in intensive care units [2]. Currently, the in-hospital sepsis and septic shock mortality remains as high as 20–30% [3]. Patients with sepsis may suffer from various degrees of heart dysfunction [4], and in severe cases, sepsis may lead to multiple organ failure [5]. Heart dysfunction in sepsis is associated with significantly increased mortality. When patients with sepsis have heart dysfunction, the mortality rate may be significantly increased by 20–50% [6]. In sepsis, the toll-like receptors (TLRs), inflammasomes, and other pattern recognition receptors would initiate the immune responses after recognizing the pathogen-associated molecular patterns derived from microorganisms [7]. Lipopolysaccharide (LPS), which is a pathogen-associated molecular pattern [8,9], is recognized by TLR4. It can trigger immune responses and act as an early signal of pathogenic microbial infection [10,11]. LPS is the main component of the cell wall of gram-negative bacteria [12] and has been widely used for inducing sepsis models [13,14]. However, there is no specific effective treatment for sepsis patients; therefore, sepsis has been mainly treated by controlling the infection with antibiotics and supporting the organ function [15,16]. In sepsis, how to reduce severe myocardial damage and the occurrence of heart dysfunction have attracted much attention.

After activation, TLR4 can induce inflammation [10,12,17,18,19] and the expression of nuclear factor kappa-B (NF-κB)-dependent pro-inflammatory cytokines, such as the tumor necrosis factor α (TNF-α) and interleukin-6 (IL-6) [20,21]. Many studies have shown that the inflammatory response induced by the TLR4 pathway plays an important role in myocardial injury caused by sepsis [22,23,24,25]. On the other hand, after the Notch receptor binds to its ligand, its transmembrane domain would be cleaved by the γ-endocrine enzyme complex to release the intracellular active form of Notch intracellular domain (NICD), which enters the nucleus to interact with target genes and induce the transcription of Notch target genes (such as the Hairy and Enhancer of Split 1 [Hes1] gene) [26,27,28]. There is crosstalk between the Notch and TLR4 signaling pathways. The activated TLR4 signal may directly regulate the Notch cascade through the histone modification at the target gene sites of Notch [29] or indirectly regulate the Notch cascade by inducing the Notch receptors and ligands [30,31]. Many studies have shown that the Notch signaling can enhance the TLR4-related inflammatory responses, both *in vitro* and *in vivo*, and the inflammatory response would be declined after inactivating the Notch signaling [29,31,32]. The NOTCH pathway can increase the expression of cytokines in the heart and strengthen the inflammatory responses in a mouse model of myocardial ischemia, but blocking the NOTCH pathway does not reduce the area of myocardial infarction [33]. However, the roles of the Notch pathways in septic myocardial injury need to be further studied.

This study investigated the roles of TLR4 and Notch signaling in septic myocardial injury and the effects of the interaction between TLR4 and Notch on septic heart tissue. The sepsis model was induced by LPS. The TLR4 and Notch signaling pathways were, respectively, blocked with (6*R*)-6-[*N*-(2-chloro-4-fluorophenyl)sulfamoyl]cyclohex-1-ene-1-carboxylate (TAK242) and *N*-[*N*-(3,5-difluorophenacetyl)-l-alanyl]-*s*-phenylglycinetbutylester (DAPT). The heart function, pathological damage, inflammation level in septic heart, and the key proteins of TLR4 and Notch signaling were evaluated. Our findings may help identify novel mechanisms for sepsis myocardial injury and cardiac dysfunction and provide therapeutic targets for the disease treatment.

## 2 Methods

### 2.1 Animals

Totally, 24 male SD rats (7–8-weeks-old; weighing 180–200 g) were provided by the Animal Experiment Center of Xinjiang Medical University. All animals were individually housed at room temperature (22–24°C) under standard conditions, with a light/dark cycle of 12:12 h. Animal experiments started after 7 days of acclimatization to the environment. All animals were subjected to free access to food and water but fasted for 12 h before the experiments.


**Ethical approval:** The research related to animal use has been complied with all the relevant national regulations and institutional policies for the care and use of animals, and were approved by the Ethics Committee of the Animal Experiment Center of Xinjiang Medical University (Ethics approval No.: IACUC20200924-21).

### 2.2 Animal modeling

The SD rats were randomly divided into control, LPS, LPS + TAK242, and LPS + DAPT groups, with eight rats in each group. In the control group, rats received an intraperitoneal injection of 10% DMSO + 90% corn oil. In the LPS group, rats were injected intraperitoneally with 10% DMSO + 90% corn oil and LPS (15 mg/kg; derived from the *Escherichia coli* [serum type: Coli O55:B5]) to induce sepsis [34]. Rats in the LPS + TAK242 group received intraperitoneal injection of TAK242 (3 mg/kg; dissolved in 10% DMSO + 90% corn oil; MedChemExpress, USA) [35] and LPS (15 mg/kg). For rats in the LPS + DAPT group, they received intraperitoneal injection of DAPT (100 mg/kg; dissolved in 10% DMSO + 90% corn oil; MedChemExpress, USA) [36] and LPS (15 mg/kg). The TAK242 and DAPT injections were conducted 3 h before LPS stimulation. At 14 h after injection, the rats were subjected to anesthesia with an intraperitoneal injection of sodium pentobarbital (1%; 40 mg/kg body weight), followed by echocardiography. Tissue specimens were collected immediately after the echocardiography.

### 2.3 Cardiac Doppler ultrasound examination

A high-frequency RMV707B high-frequency ultrasound probe (Visual Sonics, Toronto, ON, Canada) was used for the cardiac function assessment. All the images were collected by an experienced operator who was blinded to the experimental design. M-mode echocardiograms were collected from the long and short axis papillary muscles of the parasternal left heart. The left ventricular end-diastolic volume (LVEDV), left ventricular end-systolic volume (LVESV), ejection fraction (EF), and fractional shortening (FS) were calculated.

### 2.4 Western blot analysis

The heart apical tissue was lysed for protein extraction. Totally 100 μg of total protein was separated by SDS-PAGE and then electronically transferred onto the PVDF membrane. After blocking with 10% non-fat milk at room temperature for 2 h, the membrane was treated with the primary antibodies against GAPDH (1:10,000 dilution; Abcam, Cambridge, UK), NICD (1:1,000 dilution; Abcam), NF-κB P65 (1:1,000 dilution; Abcam), P-NF-κB P65 (1:1,000 dilution; Abcam), Hes1 (1:1,000 dilution; Abcam), TNF-α (1:1,000 dilution; Abcam), IL-6 (1:1,000 dilution; Affinity Biosciences, China), and caspase-3 (1:2,000 dilution; Abcam), respectively, at 4°C overnight. Then, the membrane was treated with goat anti-rabbit IgG H&L (HRP) (1:20,000, Abcam) at 37°C for 2 h. Protein bands were developed with the ECL kit (Boster, Wuhan, Hubei, China) and analyzed with the Image Lab system. GAPDH was used as an internal reference.

### 2.5 Quantitative real-time PCR

Total RNA was obtained with the RNA isolation kit (Tiangen, Beijing, China) from the apex of the heart. Then, the total RNA was subjected to reverse transcription using the FastKing RT kit (Tiangen). The quantitative real-time PCR was performed with the SuperReal PreMix Plus (SYBR Green) (Tiangen) on the Quantstudio^TM^ 6 Flex real-time PCR system (Thermo-Fisher Scientific, Waltham, MA, USA). The primers were all purchased from Bomed (Beijing, China), and the sequences were as follows: *GAPDH*, forward 5′-TTGTGCAGTGCCAGCCTC-3′ and reverse 5′-GAAGGGGTCGTTGATGGCAA-3′; *TNF-α*, forward 5′-ATGGGCTCCCTCTCATCAGT-3′ and reverse 5′-GCTTGGTGGTTTGCTACGAC-3′; and *IL-6*, forward 5′-CTTGGAAATGAGAAAAGAGTTGTGC-3′ and reverse 5′-ACGGAACTCCAGAAGACCAG-3′. PCR conditions were set as follows: 95°C for 15 min; 95°C for 10 s, and 55°C for 30 s, for 40 cycles. The 2^−DDCT^ method was used to analyze the relevant expression levels of target genes. GAPDH was used as an internal reference.

### 2.6 H&E staining

The heart tissues were fixed with 4% paraformaldehyde for 48 h and then embedded in paraffin. After deparaffinization, the tissues were made into sections and stained with hematoxylin and eosin (H&E) according to the routine procedure. The degree of inflammatory cell infiltration and cardiomyocyte damage was assessed.

### 2.7 Immunohistochemistry

The deparaffinized tissue sections were incubated with 3% H_2_O_2_ at room temperature for the immunohistochemical staining. After blocking with goat serum, the sections were treated with the primary antibodies against NICD (1:400 dilution; Abcam), NF-κB P65 (1:400 dilution; Abcam), and Hes1 (1:400 dilution; Abcam), respectively, overnight. Then, the sections were incubated with secondary antibodies (1:20,000 dilution; Abcam), followed by the DAB development and the subsequent hematoxylin staining. Sections were observed under the microscope.

### 2.8 TUNEL assay

The apoptosis in heart tissue sections was assessed using TUNEL Apoptosis Detection Kit Ⅲ-FITC (BOSTER, Wuhan, China) according to the instructions. The images were observed under a microscope (Olympus, Tokyo, Japan). The bright yellow-green spots represent the positive results of apoptosis.

### 2.9 Statistical analysis

Data are expressed as mean value ± SD. The SPSS 25.0 software (SPSS, Chicago, IL, USA) was used for statistical analysis. The ANOVA followed by Tukey’s test was performed for data comparison. *P* < 0.05 was considered statistically significant.

## 3 Results

### 3.1 Blocking TLR4 reduces cardiac dysfunction and myocardial injury in LPS-induced sepsis

**Figure 1:**
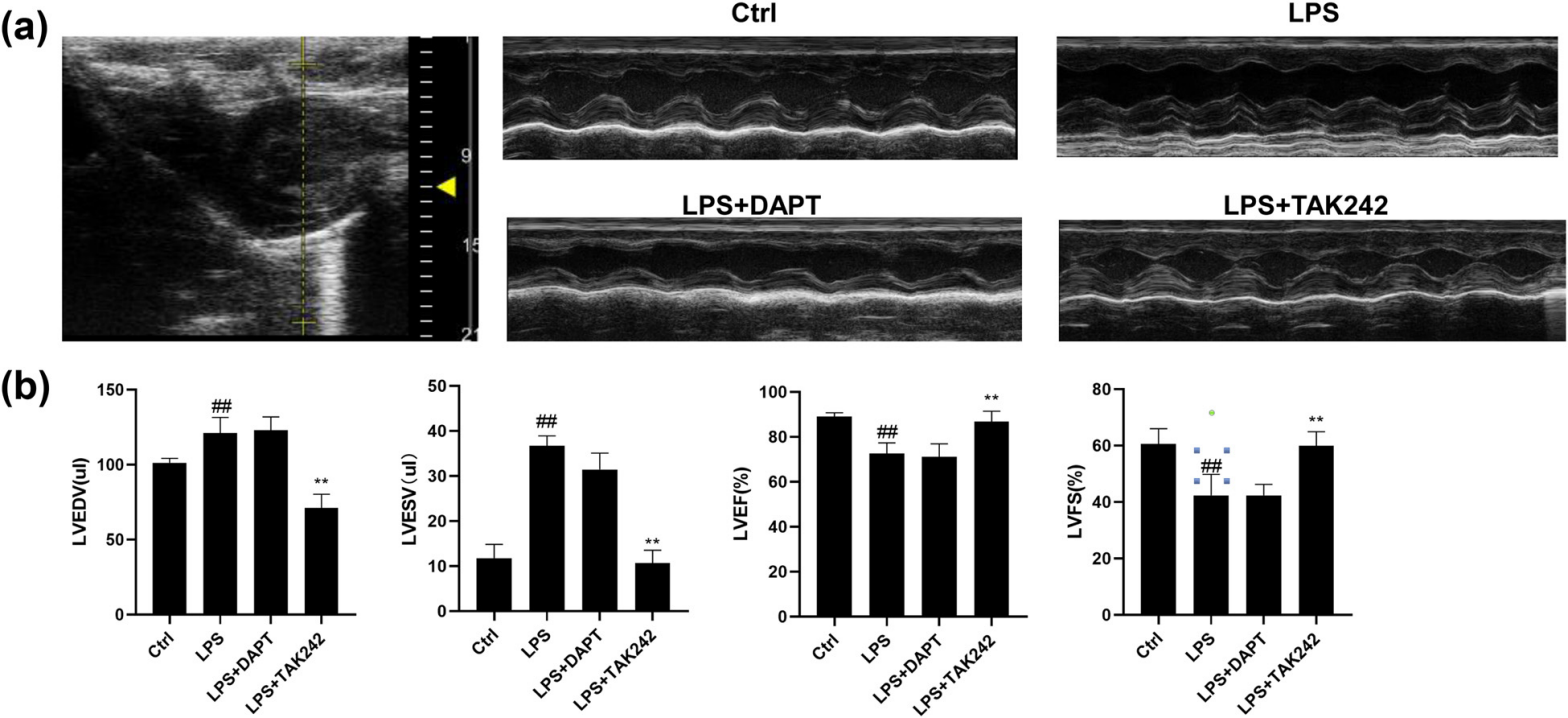
Analysis of heart function. (a) The large picture on the left panel is a representative picture of the position of the heart under echocardiography, located in the left ventricle. The four pictures on the right are representative echocardiography pictures of rats in the control, LPS, LPS + DAPT, and LPS + TAK242 groups. (b) Histogram of LVEDV, LVESV, EF, and FS measured by echocardiography. Compared with control, ^#^ *P* < 0.05, ^##^ *P* < 0.01; and compared with LPS group, ^*^ *P* < 0.05, ^**^ *P* < 0.01.

Cardiac Doppler ultrasound was performed to evaluate the cardiovascular function of rats in each group. Our results showed that, in the rats from the LPS group, the LVEDV and LVESV were significantly higher, while the EF and FS of the rats in the LPS group were significantly lower than the control group and the LPS + TAK242 group (Figure 1). There were no significant differences in LVEDV, LVESV, EF, and FS between the LPS + DAPT group and the LPS group (Figure 1). These results suggest that there is heart dysfunction in the rat model with sepsis. Moreover, blocking the TLR4 pathway with TAK242 could significantly improve heart dysfunction, while this effect was not observed after blocking the Notch pathway with DAPT.

**Figure 2:**
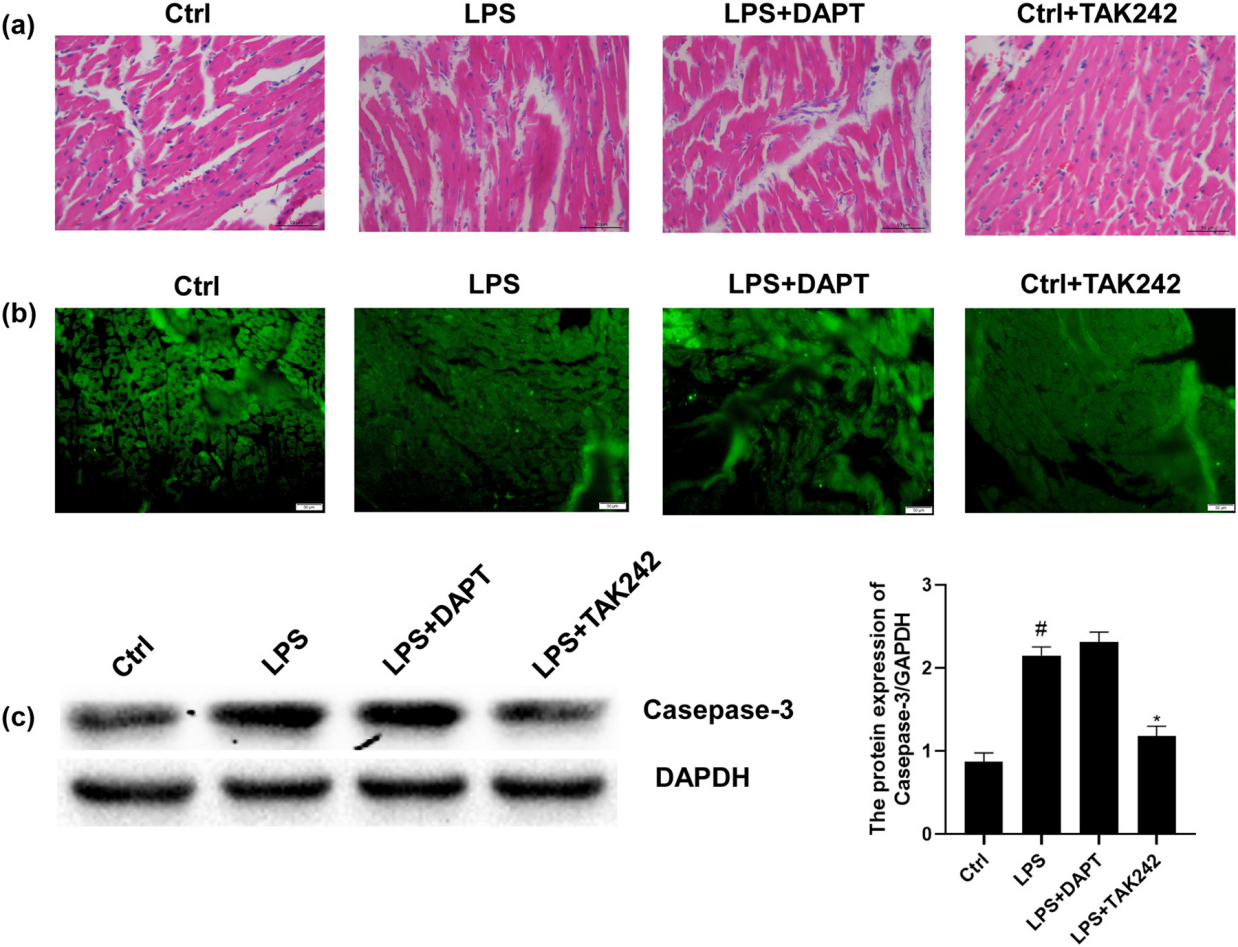
Analysis of histopathological changes and casepase-3. (a) Myocardial H&E staining (×400). (b) TUNEL apoptosis Detection (×400). (c) Expression levels of caspase-3 were detected with the Western blot analysis. Compared with control, ^#^ *P* < 0.05; and compared with LPS group, ^*^ *P* < 0.05.

To detect the pathological changes in the heart of rats with sepsis, the H&E staining was performed. Compared with the control group, rats in the LPS group had more obvious myocardial interstitial edema, disordered myocardial fiber arrangement, cardiomyocyte edema, nuclear necrosis, and obvious inflammatory cell infiltration (Figure 2a). Compared with the LPS group, the myocardial fibers in rats from the LPS + TAK242 group were arranged more neatly, the inflammatory cell infiltration was reduced, and the nuclear necrosis was improved. Compared with the LPS group, the LPS + DAPT group had less inflammatory cell infiltration, but the degeneration and necrosis of cardiomyocytes were not improved. These results indicate that sepsis could cause myocardial tissue damage, which could be reduced by TAK242 but not DAPT.

Moreover, TUNEL staining showed that the apoptosis rate of the LPS group was higher than that of the control group (Figure 2b). The apoptosis rate of the LPS + DAPT group was not evidently different from the LPS group. However, the apoptosis rate of the LPS + TAK242 group was lower than that of the LPS group. Western blot analysis showed that the expression levels of the caspase-3 in the LPS group were significantly higher than in the control group and the LPS + TAK242 group (Figure 2c). There was no significant difference in caspase-3 between the LPS group and the LPS + DAPT group, indicating that sepsis may increase the apoptosis of cardiomyocytes. TAK242, but not DAPT, could reduce cardiomyocyte apoptosis caused by sepsis.

Together, blocking the TLR4 pathway by TAK242 may have protective effects on the septic myocardium, while the protective effects were not observed after blocking the Notch pathway with DAPT.

### 3.2 Blocking TLR4 or NOTCH reduces inflammatory responses in the heart in LPS-induced sepsis

**Figure 3:**
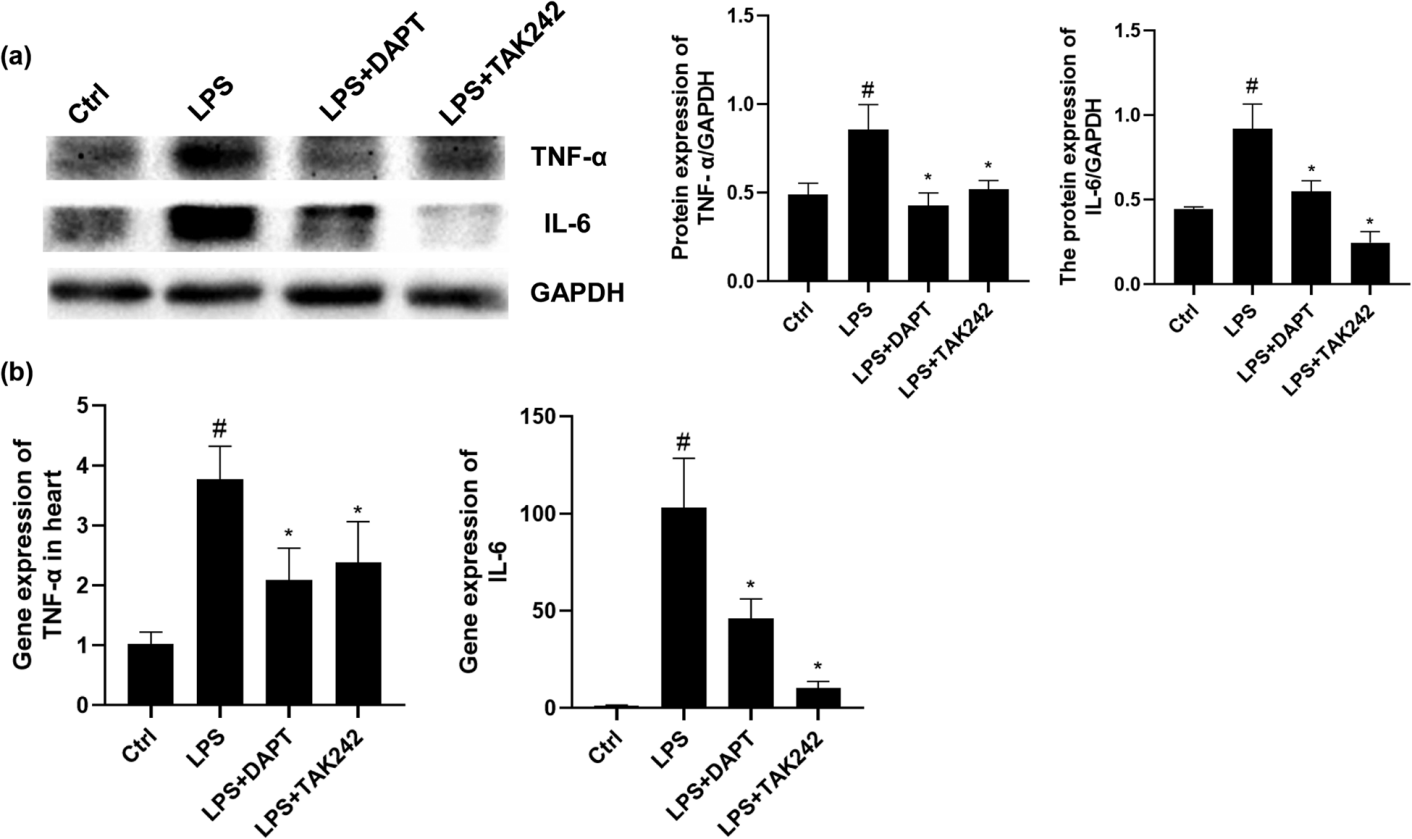
Analysis of cytokine levels. (a) Expression levels of TNF-α and IL-6 were detected with the Western blot analysis. (b) The mRNA expression levels of *TNF-α* and *IL-6* were detected with quantitative real-time PCR. Compared with control, ^#^ *P* < 0.05; and compared with LPS group, ^*^ *P* < 0.05.

To detect the levels of local inflammation in the heart tissue, the mRNA and protein expression levels of IL-6 and TNF-α in the heart tissue were assessed. Our results showed that, compared with the control group, the IL-6 and TNF-α mRNA and protein expression levels in the myocardium were significantly increased when the rats were stimulated with LPS (Figure 3a and b). Moreover, compared with the LPS group, the TAK242 and DAPT intervention significantly reduced the mRNA and gene expression levels of IL-6 and TNF-α in the myocardium. These findings suggest that the TLR4 and Notch signaling pathways may jointly regulate the release of inflammatory cytokines in the septic heart, and the degree of septic myocardial damage may be related to these inflammatory factors.

### 3.3 LPS-induced sepsis activates NOTCH, and blocking TLR4 reduces activated NOTCH

**Figure 4:**
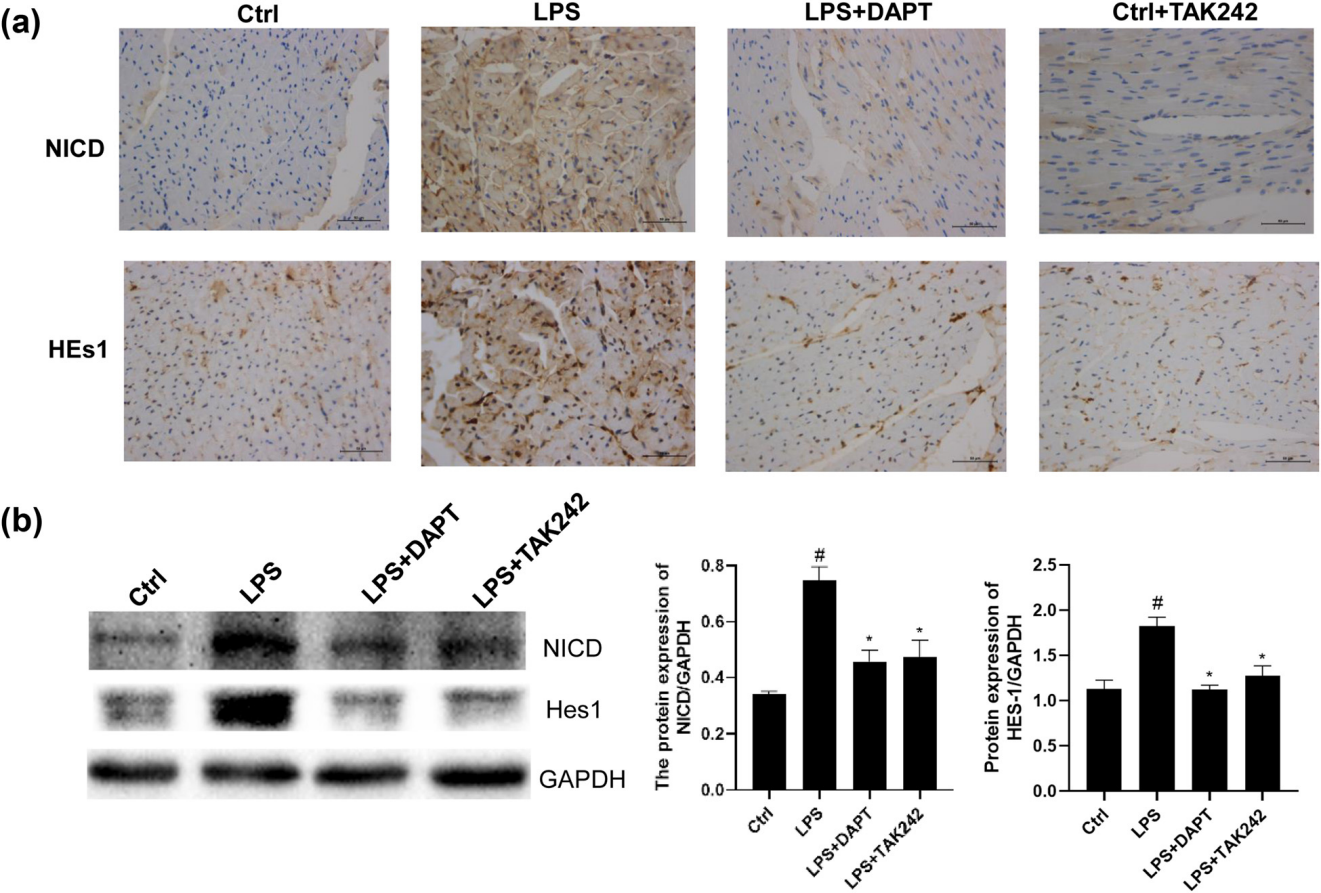
Analysis of NICD and Hes1 proteins in NOTCH signaling pathway. (a) Expressions of NICD and Hes1 were detected with immunohistochemistry (×400). (b) Expression levels of NICD and Hes1 were detected with the Western blot analysis. Compared with control, ^#^ *P* < 0.05; and compared with LPS group, ^*^ *P* < 0.05.

Both immunohistochemistry (Figure 4a) and Western blot (Figure 4b) results showed that the contents of NICD and Hes1 were increased after the LPS stimulation, indicating that the LPS stimulation can activate the Notch signaling pathway in the myocardium. After the DAPT administration, the contents of NICD and Hes1 were decreased, indicating the success of the DAPT intervention. Moreover, compared with the LPS group, TAK242 intervention reduced the protein expression levels of NICD and Hes1, indicating that the activation of Notch signal in septic myocardium is related to the activation of TLR4 signal.

### 3.4 Blocking TLR4 or NOTCH inhibits NF-κB nuclear translocation and activation in LPS-induced sepsis

**Figure 5:**
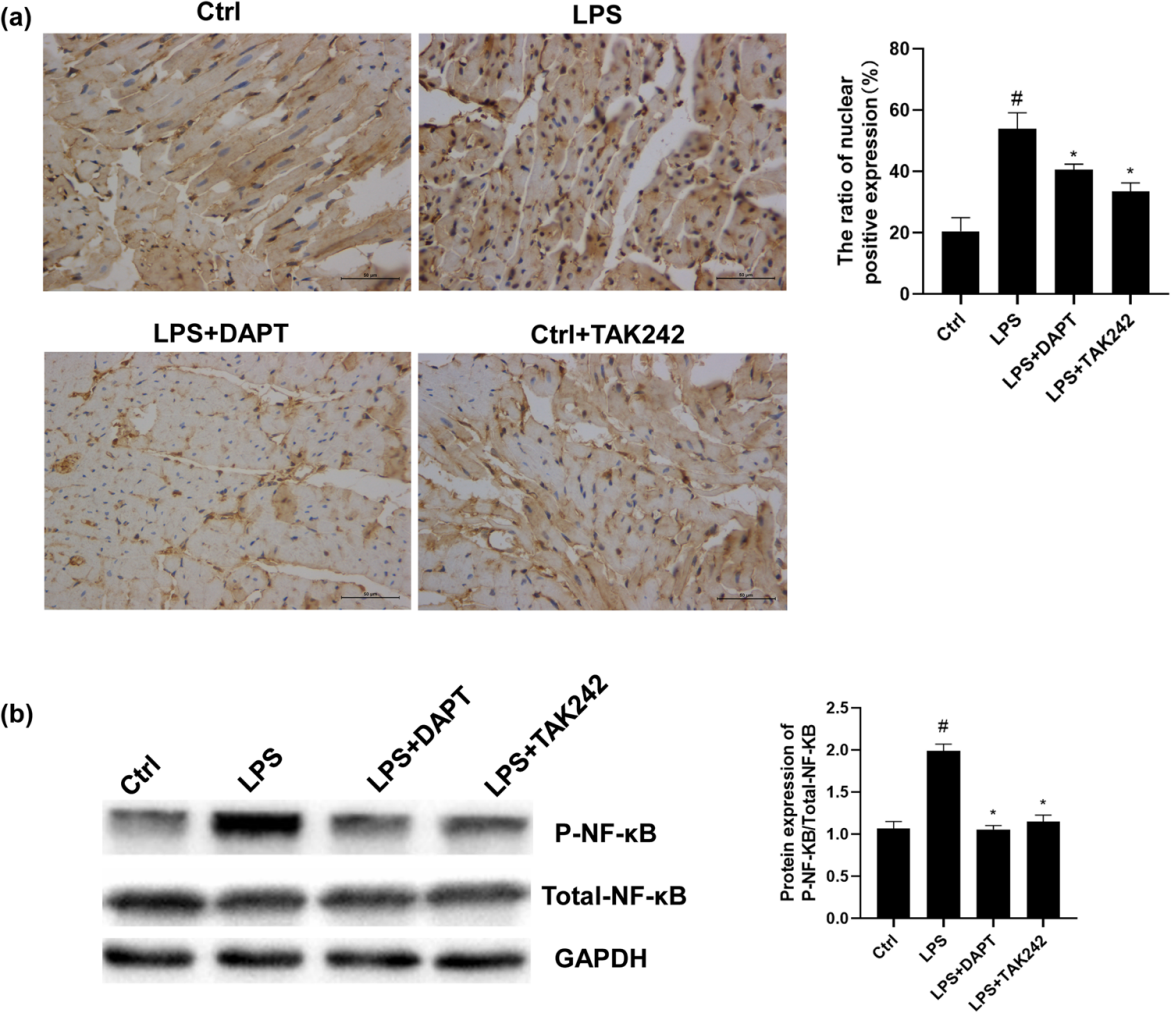
Analysis of NF-κB protein. (a) Nuclear translocation of NF-κB in rat myocardium was detected with the immunohistochemistry. The bar graph represents the ratio of the number of positive nuclei to the total number of nuclei as shown by immunohistochemistry. The larger the ratio, the higher the degree of nuclear translocation (×400). (b) The phosphorylation of NF-κB in rat myocardial tissue was detected with the Western blot analysis. Compared with control, ^#^ *P* < 0.05; and compared with LPS group, ^*^ *P* < 0.05.

Our results by immunohistochemistry (Figure 5a) and Western blot (Figure 5b) demonstrated that the LPS stimulation increased the nuclear translocation of NF-κB and increased the P-NF-κB/NF-κB ratio in the myocardium. Compared with the LPS group, NF-κB nuclear translocation was decreased, and the P-NF-κB/NF-κB ratios were declined, after the TAK242 intervention, indicating that the TLR4 pathway may act through the NF-κB pathway in the septic myocardium. Compared with the LPS group, the NF-κB nuclear translocation was decreased, and the P-NF-κB/NF-κB ratio was reduced when DAPT was given, indicating that the Notch signaling may increase NF-κB phosphorylation and nuclear translocation to regulate the inflammatory response induced by the TLR4-NF-κB pathway.

## 4 Discussion

In this study, our results showed that the LPS stimulation could activate the TLR4 and Notch signaling pathways in the heart tissue, and the mutual crosstalk relationship between the TLR4-NF-κB pathway and the Notch pathway could jointly regulate the inflammatory response in the septic heart. The TLR4 pathway activated by LPS could enhance the activation of the Notch pathway, which could further enhance the activation of the TLR4-NF-κB pathway, thereby enhancing the inflammatory response mediated by TLR4. Moreover, the Notch signaling may activate NF-κB to enhance the inflammatory responses. Blocking the TLR4 pathway with TAK242 effectively prevented heart dysfunction and myocardial damage in sepsis rats, which was not observed with DAPT.

Studies have shown that the activation of TLR4 signaling in severe sepsis can enhance the inflammatory response by promoting the release of pro-inflammatory cytokines, causing heart damage, and inhibiting heart function [22,24]. Moreover, the TLR4 expressed by cardiomyocytes plays an important role in the acute phase of septic heart dysfunction [25]. On the contrary, the activation of TLR4 can increase the expression of G protein-coupled receptor kinases (GRK2) [37]. GRK2 can lead to the phosphorylation of β2-adrenergic receptors (β2AR), and the phosphorylated β2AR can inhibit the activation of NF-κB through β-arrestins to suppress the inflammatory response [38]. In a mouse model of myocardial infarction with knockdown of β1-adrenergic receptors (β1AR), the inflammatory response was enhanced after GRK2 inhibition. However, β2AR-mediated cardiac contractility was found to be enhanced, β2AR anti-apoptotic signaling was enhanced, and mouse survival was increased [39]. Therefore, the TLR4 signaling pathway can promote the inflammatory response through NF-κB to mediate cardiac injury. Meanwhile, the TLR4 signaling pathway can also increase the expression of GRK2, and GRK2 can inhibit the activation of NF-κB through the β2AR-β-arrestins pathway to reduce the inflammatory response. TAK242 can selectively inhibit the TLR4 signaling pathway and reduce the release of various inflammatory factors in sepsis, thereby reducing the mortality of sepsis [40,41]. TAK-242, a specific inhibitor of TLR4 signaling, can inhibit MyD88 and TRIF-dependent pathways by binding to Cys747 in the intracellular domain of TLR4 [42,43]. Previous studies have shown that TAK-242 prevented acute kidney injury and lung injury in LPS-injected sheep and mice [44,45]. TAK242 can protect against LPS-induced cardiac dysfunction and myocardial injury by blocking the TLR4-mediated inflammatory response [46]. Consistently, in this study, after using TAK242 to block the TLR4 signaling pathway in septic rats, the activation and nuclear translocation of NF-κB were reduced, the contents of inflammatory factors (TNF-α and IL-6) were reduced, and the heart damage was improved, indicating that the activation of the TLR4 signaling pathway in sepsis could promote the inflammatory response through the NF-kB pathway, and lead to myocardial damage and heart dysfunction.

In terms of the TLR-mediated regulation of Notch signaling, TLRs may indirectly regulate the Notch signaling by inducing the expression of Notch receptors and ligands, thereby activating the Notch signaling pathway. Many studies have reported increased expression levels of Notch receptors and ligands after activation of TLRs [29,30,31,47]. A previous study indicated that TLR4 signaling pathway activation resulted in an elevation of DLL4 expression through ERK/FOXC2 signaling pathway [48]. Another study showed that TLR4–NF-κB signaling induced hepatocyte Jag1 expression and triggered inter-hepatocyte Jagged1/Notch signaling [49]. The Notch signaling pathway regulates the differentiation, proliferation, survival, and development of cells [50]. In addition, the Notch signaling regulates the production of cytokines in T lymphocytes and macrophages [29,31]. However, it is not clear whether Notch is involved in the enhancement of inflammatory response and myocardial damage in septic heart tissue. Our results showed that the content of NICD was increased after LPS activated the TLR4 pathway. When TAK242 was used to block the TLR4 pathway, the content of NICD was decreased, indicating that the Notch activation in the septic heart may be related to the activation of TLR4 signal.

It is shown that the activation of Notch signals can promote the differentiation of most immune cells to a pro-inflammatory phenotype, thereby increasing the inflammatory responses [51,52]. In the rat myocardial infarction model, the inhibition of Notch signal could reduce the differentiation of macrophages into M1 macrophages, thereby reducing the levels of inflammatory factors and reducing the inflammatory responses [33]. These findings have shown that Notch signaling can promote inflammatory responses. DAPT is often used as a specific inhibitor of γ-secretase and a blocking agent of Notch pathway [53]. DAPT inhibits the formation of the soluble NICD protein by preventing the cleavage of γ-secretase at the S3 site of the Notch receptor [54,55]. In this study, to verify the role of the Notch1 signaling pathway in the inflammatory response induced by TLR4 in the heart, the γ-secretase inhibitor DAPT was used to inhibit the activation of Notch in the septic rats. After DAPT intervention, the inflammatory factors (TNF-α and IL-6) were decreased, indicating that the inhibition of Notch signal would reduce the inflammatory response of the heart tissue. Therefore, the activation of Notch signal in sepsis may play an important role in the enhancement of the inflammatory response induced by TLR4 in the heart.

Our results showed that the Notch signaling had an important effect on the activation of NF-κB in the heart. The DAPT was used to inhibit the Notch signaling pathway during LPS stimulation, which attenuated the phosphorylation and nuclear translocation of NF-κB p65. Studies have shown that the Notch1 signaling pathway in macrophages can interact with NF-κB to enhance the inflammatory responses induced by TLR4 [56,57]. Moreover, in the interstitial cells of human aortic valve stenosis, Notch1 enhances the inflammatory response and promotes the osteogenic response under the stimulation of TLR4 by regulating the activation of NF-κB [58,59]. NOTCH3 is the only NOTCH receptor expressed in resting macrophages [60]. In macrophages isolated from patients with atherosclerosis, NOTCH1 appears to play a more prominent role than those of NOTCH2 and NOTCH3 in the regulation of the NF-κB signaling pathway and in the induction of pro-inflammatory gene expression [61]. On the contrary, NOTCH4 exhibits anti-inflammatory activity in activated macrophages by interfering with interferon-gamma, TLR4 signaling, and NF-kB transcriptional activity [62]. Notch signaling can trigger free NF-κB to translocate into the nucleus and stimulate the NF- κB pathway, leading to the activation of pro-inflammatory cytokine genes and causing inflammatory cytokine secretion [48,63]. After NF-κB is activated, the p50-p65 forms a heterodimer and undergoes nuclear translocation, which triggers the transcription of mRNA encoding a series of mediators, such as adhesion molecules, cytokines, chemokines, and procoagulants [64]. These findings suggest that the Notch signaling pathway plays an important role in NF-κB-mediated inflammation.

In this study, however, when DAPT was used to block the Notch pathway, the septic heart dysfunction and myocardial damage showed no obvious improvement. The possible reason would be that Notch could mediate inflammation and may also have repairing effects. Previous findings indicated that the activation of Notch signaling pathway played an important role in the regeneration of endocardium injury, which may play a complex role in the interaction with different signaling molecules [65]. Blocking the Notch pathway in the infarct model could reduce the infiltration of inflammatory cells and the inflammatory responses in the heart, which, however, did not reduce the area of the heart infarction but increased the infarction area [33]. On the other hand, Notch is crucial in promoting the survival of cardiomyocytes and endothelial cells and maintaining the contractile phenotype of VSMCs [66]. In the cardiovascular system, Notch activation prevents apoptosis of cardiomyocytes [67] and endothelial cells caused by different types of insults [68]. Thus, activation of Notch in the heart [69] and endothelium [70] could represent a new therapeutic approach against diseases such as coronary artery disease and heart failure [71]. It has been demonstrated in mouse models that Notch signals over-expressed in vascular, stromal cells could significantly improve the repairing ability of mesenchymal stem cells [72]. Notch signal plays an important role in the repairing of vascular endothelial injury [73]. Notch1 and Jagged1, expressed in the adult heart, can protect cardiac tissue under pathophysiological conditions [72]. Notch1 signaling is activated following myocardial injury by repressing reactive oxygen species production and by stabilizing mitochondrial membrane potential [74]. Notch1 signaling can attenuate myocardial ischemia/reperfusion injury by suppressing oxidative/nitrative stress [75]. These findings suggest the damage repairing effects of the Notch signal. However, the repairing mechanism of Notch in septic heart injuries needs further verification.

## 5 Conclusion

**Figure 6:**
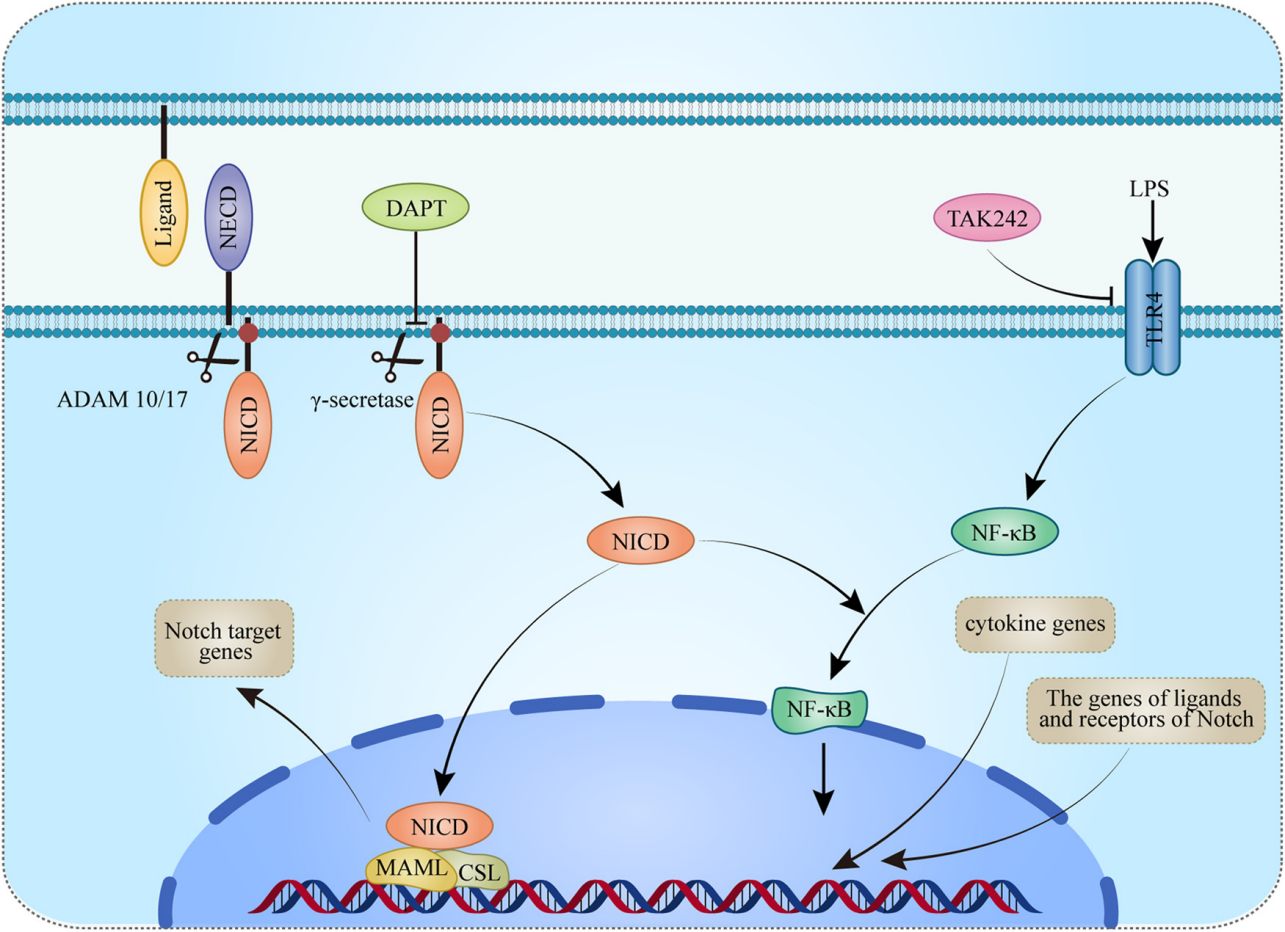
A schematic figure illustrating the interaction between TLR4 and Notch1 signaling pathways. The TLR4 receptor activated by LPS promotes the expression of inflammatory factors and the expression of Notch receptors and ligands through the NF-KB pathway. The increase in the expression of Notch receptors and ligands promotes the activation of the Notch signaling pathway. After activation, the Notch intra-cellular domain is released into the cytoplasm, which enhances the inflammatory response by promoting the activation of NF-KB. This study used TAK242 to block the TLR4 pathway and DAPT to block the Notch pathway. The relationship between the two pathways was investigated.

In conclusion, our results showed the interaction between TLR4 and Notch signaling pathways may enhance the inflammatory response in septic rat hearts by regulating the activation of NF-κB (Figure 6). Blocking the TLR4 pathway with TAK242 could improve heart dysfunction and myocardial damage in sepsis. Blocking the Notch pathway with DAPT could not effectively prevent heart dysfunction and myocardial damage in rats with sepsis. The possible reason is that Notch can mediate inflammation and may also have repairing effects. Further studies are warranted for verification.

## References

[1] Singer M, Deutschman CS, Seymour CW, Shankar-Hari M, Annane D, Bauer M, et al. The third international consensus definitions for sepsis and septic shock (Sepsis-3). JAMA. 2016;315:801–1010. 10.1001/jama.2016.0287 PMC4968574 26903338

[2] Jentzer JC, Vallabhajosyula S, Khanna AK, Chawla LS, Busse LW, Kashani KB. Management of refractory vasodilatory shock. Chest. 2018;154:416–26. 10.1016/j.chest.2017.12.021 29329694

[3] Kotecha AA, Vallabhajosyula S. Clinical outcomes of weight-based norepinephrine dosing in underweight and morbidly obese patients: a propensity-matched analysis. J Intensive Care Med. 2020;35:554–61. 10.1177/0885066618768180 29628015

[4] Vieillard-Baron A, Cecconi M. Understanding cardiac failure in sepsis. Intensive Care Med. 2014;40:1560–3. 10.1007/s00134-014-3367-8 24966063

[5] Chong J, Dumont T, Francis-Frank L, Balaan M. Sepsis and septic shock: a review. Crit Care Nurs Q. 2015;38:111–20. 10.1097/CNQ.0000000000000052 25741952

[6] Geri G, Vignon P, Aubry A, Fedou AL, Charron C, Silva S, et al. Cardiovascular clusters in septic shock combining clinical and echocardiographic parameters: a post hoc analysis. Intensive Care Med. 2019;45:657–67. 10.1007/s00134-019-05596-z 30888443

[7] Wiersinga WJ, Leopold SJ, Cranendonk DR, van der Poll T. Host innate immune responses to sepsis. Virulence. 2014;5:36–44. 10.4161/viru.25436 PMC3916381 23774844

[8] Xu T, Deng R, Li X, Zhang Y, Gao MQ. RNA-seq analysis of different inflammatory reactions induced by lipopolysaccharide and lipoteichoic acid in bovine mammary epithelial cells. Microb Pathog. 2019;130:169–77. 10.1016/j.micpath.2019.03.015 30878619

[9] Tsugami Y, Wakasa H, Kawahara M, Nishimura T, Kobayashi K. Lipopolysaccharide and lipoteichoic acid influence milk production ability via different early responses in bovine mammary epithelial cells. Exp Cell Res. 2021;400:112472. 10.1016/j.yexcr.2021.112472 33450209

[10] Fu Y, Zhou E, Liu Z, Li F, Liang D, Liu B, et al. Staphylococcus aureus and Escherichia coli elicit different innate immune responses from bovine mammary epithelial cells. Vet Immunol Immunopathol. 2013;155:245–52. 10.1016/j.vetimm.2013.08.003 24018311

[11] Park BS, Lee JO. Recognition of lipopolysaccharide pattern by TLR4 complexes. Exp Mol Med. 2013;45:e66. 10.1038/emm.2013.97 PMC3880462 24310172

[12] Beutler B. TLR4 as the mammalian endotoxin sensor. Curr Top Microbiol Immunol. 2002;270:109–20. 10.1007/978-3-642-59430-4_7 12467247

[13] Schultz MJ, van der Poll T. Animal and human models for sepsis. Ann Med. 2002;34:573–81. 10.1080/078538902321117797 12553497

[14] Fink MP, Heard SO. Laboratory models of sepsis and septic shock. J Surg Res. 1990;49:186–96. 10.1016/0022-4804(90)90260-9 2199735

[15] Rhodes A, Evans LE, Alhazzani W, Levy MM, Antonelli M, Ferrer R, et al. Surviving sepsis campaign: international guidelines for management of sepsis and septic shock: 2016. Intensive Care Med. 2017;43:304–77. 10.1007/s00134-017-4683-6 28101605

[16] Levy MM, Evans LE, Rhodes A. The surviving sepsis campaign bundle: 2018 update. Intensive Care Med. 2018;44:925–8. 10.1007/s00134-018-5085-0 29675566

[17] Poltorak A, He X, Smirnova I, Liu MY, Van Huffel C, Du X, et al. Defective LPS signaling in C3H/HeJ and C57BL/10ScCr mice: mutations in Tlr4 gene. Science. 1998;282:2085–8. 10.1126/science.282.5396.2085 9851930

[18] Beutler B, Du X, Poltorak A. Identification of Toll-like receptor 4 (Tlr4) as the sole conduit for LPS signal transduction: genetic and evolutionary studies. J Endotoxin Res. 2001;7:277–80. 11717581

[19] Cao C, Chai Y, Shou S, Wang J, Huang Y, Ma T. Toll-like receptor 4 deficiency increases resistance in sepsis-induced immune dysfunction. Int Immunopharmacol. 2018;54:169–76. 10.1016/j.intimp.2017.11.006 29149705

[20] Frantz S, Ertl G, Bauersachs J. Mechanisms of disease: Toll-like receptors in cardiovascular disease. Nat Clin Pract Cardiovasc Med. 2007;4:444–54. 10.1038/ncpcardio0938 17653117

[21] Nakayama Y, Fujiu K, Yuki R. A long noncoding RNA regulates inflammation resolution by mouse macrophages through fatty acid oxidation activation. Proc Natl Acad Sci USA. 2020;117:14365–75. 10.1073/pnas.2005924117 PMC7322040 32513690

[22] Zhou D, Zhu Y, Ouyang MZ, Zhang M, Tang K, Niu CC, et al. Knockout of Toll-like receptor 4 improves survival and cardiac function in a murine model of severe sepsis. Mol Med Rep. 2018;17:5368–75. 10.3892/mmr.2018.8495 29393431

[23] Rameshrad M, Maleki-Dizaji N, Vaez H, Soraya H, Nakhlband A, Garjani A. Lipopolysaccharide induced activation of toll like receptor 4 in isolated rat heart suggests a local immune response in myocardium. Iran J Immunol. 2015;12:104–16. 10.22034/iji.2015.16740 26119193

[24] Avlas O, Fallach R, Shainberg A, Porat E, Hochhauser E. Toll-like receptor 4 stimulation initiates an inflammatory response that decreases cardiomyocyte contractility. Antioxid Redox Signal. 2011;15:1895–909. 10.1089/ars.2010.3728 21126202

[25] Fallach R, Shainberg A, Avlas O, Fainblut M, Chepurko Y, Porat E, et al. Cardiomyocyte Toll-like receptor 4 is involved in heart dysfunction following septic shock or myocardial ischemia. J Mol Cell Cardiol. 2010;48:1236–44. 10.1016/j.yjmcc.2010.02.020 20211628

[26] Kopan R, Ilagan MX. The canonical Notch signaling pathway: unfolding the activation mechanism. Cell. 2009;137:216–33. 10.1016/j.cell.2009.03.045 PMC2827930 19379690

[27] Kovall RA, Blacklow SC. Mechanistic insights into Notch receptor signaling from structural and biochemical studies. Curr Top Dev Biol. 2010;92:31–71. 10.1016/S0070-2153(10)92002-4 20816392

[28] Bray SJ. Notch signalling in context. Nat Rev Mol Cell Biol. 2016;17:722–35. 10.1038/nrm.2016.94 27507209

[29] Hu X, Chung AY, Wu I, Foldi J, Chen J, Ji JD, et al. Integrated regulation of Toll-like receptor responses by Notch and interferon-gamma pathways. Immunity. 2008;29:691–703. 10.1016/j.immuni.2008.08.016 PMC2585039 18976936

[30] Monsalve E, Pérez MA, Rubio A, Ruiz-Hidalgo MJ, Baladrón V, García-Ramírez JJ, et al. Notch-1 up-regulation and signaling following macrophage activation modulates gene expression patterns known to affect antigen-presenting capacity and cytotoxic activity. J Immunol. 2006;176:5362–73. 10.4049/jimmunol.176.9.5362 16622004

[31] Palaga T, Buranaruk C, Rengpipat S, Fauq AH, Golde TE, Kaufmann SH, et al. Notch signaling is activated by TLR stimulation and regulates macrophage functions. Eur J Immunol. 2008;38:174–83. 10.1002/eji.200636999 18085664

[32] Tsao PN, Wei SC, Huang MT, Lee MC, Chou HC, Chen CY, et al. Lipopolysaccharide-induced Notch signaling activation through JNK-dependent pathway regulates inflammatory response. J Biomed Sci. 2011;18:56. 10.1186/1423-0127-18-56 PMC3176188 21843347

[33] Yin J, Hu H, Li X, Xue M, Cheng W, Wang Y, et al. Inhibition of Notch signaling pathway attenuates sympathetic hyperinnervation together with the augmentation of M2 macrophages in rats post-myocardial infarction. Am J Physiol Cell Physiol. 2016;310:C41–53. 10.1152/ajpcell.00163.2015 26491050

[34] Poon BY, Raharjo E, Patel KD, Tavener S, Kubes P. Complexity of inducible nitric oxide synthase: cellular source determines benefit versus toxicity. Circulation. 2003;108:1107–12. 10.1161/01.CIR.0000086321.04702.AC 12925459

[35] Samarpita S, Kim JY, Rasool MK, Kim KS. Investigation of toll-like receptor (TLR) 4 inhibitor TAK-242 as a new potential anti-rheumatoid arthritis drug. Arthritis Research & Therapy. 2020;22:16. 10.1186/s13075-020-2097-2 PMC6979396 31973752

[36] Long J, Yang C. Notch signaling protects CD4 T cells from STING-mediated apoptosis during acute systemic inflammation. Science Advances. 2020;6(39):eabc5447. 10.1126/sciadv.abc5447 PMC7531880 32967837

[37] Ge XY, Fang SP, Zhou M, Luo J, Wei J, Wen XP, et al. TLR4-dependent internalization of CX3CR1 aggravates sepsis-induced immunoparalysis. Am J Transl Res. 2016;8:5696–705. PMC5209520 28078040

[38] Drosatos K, Lymperopoulos A, Kennel PJ, Pollak N, Schulze PC, Goldberg IJ. Pathophysiology of sepsis-related cardiac dysfunction: driven by inflammation, energy mismanagement, or both? Curr Heart Fail Rep. 2015;12:130–40. 10.1007/s11897-014-0247-z PMC4474734 25475180

[39] Salazar NC, Vallejos X, Siryk A, Rengo G, Cannavo A, Liccardo D, et al. GRK2 blockade with βARKct is essential for cardiac β2-adrenergic receptor signaling towards increased contractility. Cell Commun Signal. 2013;11:64. 10.1186/1478-811X-11-64 PMC3846709 23984976

[40] Ii M, Matsunaga N, Hazeki K, Nakamura K, Takashima K, Seya T, et al. A novel cyclohexene derivative, ethyl (6R)-6-[N-(2-Chloro-4-fluorophenyl)sulfamoyl]cyclohex-1-ene-1-carboxylate (TAK-242), selectively inhibits toll-like receptor 4-mediated cytokine production through suppression of intracellular signaling. Mol Pharmacol. 2006;69:1288–95. 10.1124/mol.105.019695 16373689

[41] Sha T, Sunamoto M, Kitazaki T, Sato J, Ii M, Iizawa Y. Therapeutic effects of TAK-242, a novel selective Toll-like receptor 4 signal transduction inhibitor, in mouse endotoxin shock model. Eur J Pharmacol. 2007;571:231–9. 10.1016/j.ejphar.2007.06.027 17632100

[42] Takashima K, Matsunaga N, Yoshimatsu M, Hazeki K, Kaisho T, Uekata M, et al. Analysis of binding site for the novel small-molecule TLR4 signal transduction inhibitor TAK-242 and its therapeutic effect on mouse sepsis model. Br J Pharmacol. 2009;157:1250–62. 10.1111/j.1476-5381.2009.00297.x PMC2743844 19563534

[43] Kawamoto T, Ii M, Kitazaki T, Iizawa Y, Kimura H. TAK-242 selectively suppresses Toll-like receptor 4-signaling mediated by the intracellular domain. Eur J Pharmacol. 2008;584:40–8. 10.1016/j.ejphar.2008.01.026 18299127

[44] Fenhammar J, Rundgren M, Forestier J, Kalman S, Eriksson S, Frithiof R. Toll-like receptor 4 inhibitor TAK-242 attenuates acute kidney injury in endotoxemic sheep. Anesthesiology. 2011;114:1130–7. 10.1097/ALN.0b013e31820b8b44 21394006

[45] Seki H, Tasaka S, Fukunaga K, Shiraishi Y, Moriyama K, Miyamoto K, et al. Effect of Toll-like receptor 4 inhibitor on LPS-induced lung injury. Inflamm Res. 2010;59:837–45. 10.1007/s00011-010-0195-3 20387088

[46] Liu Z, Yang K, Deng T, Peng P. Protective effect of TAK242 blocking Toll-like receptor 4 pathway on septic myocardial injury and cardiac dysfunction. Zhonghua Wei Zhong Bing Ji Jiu Yi Xue. 2021;33:1226–31. 10.3760/cma.j.cn121430-20210620-00915 34955133

[47] Foldi J, Chung AY, Xu H, Zhu J, Outtz HH, Kitajewski J, et al. Autoamplification of Notch signaling in macrophages by TLR-induced and RBP-J-dependent induction of Jagged1. J Immunol. 2010;185:5023–31. 10.4049/jimmunol.1001544 PMC3010732 20870935

[48] Xu D, Xia N, Hou K, Li F, Chen S, Hu Y, et al. Clematichinenoside facilitates recovery of neurological and motor function in rats after cerebral ischemic injury through inhibiting notch/NF-κB pathway. J Stroke Cerebrovasc Dis. 2019;28:104288. 10.1016/j.jstrokecerebrovasdis.2019.07.004 31395423

[49] Yu J, Zhu C. Hepatocyte TLR4 triggers inter-hepatocyte Jagged1/Notch signaling to determine NASH-induced fibrosis. Sci Transl Med. 2021;13(599):eabe1692. 10.1126/scitranslmed.abe1692 PMC8792974 34162749

[50] Fortini ME. Notch signaling: the core pathway and its posttranslational regulation. Dev Cell. 2009;16:633–47. 10.1016/j.devcel.2009.03.010 19460341

[51] Christopoulos PF, Gjolberg TT, Kruger S, Haraldsen G, Andersen JT, Sundlisaeter E. Targeting the Notch signaling pathway in chronic inflammatory diseases. Front Immunol. 2021;12:668207. 10.3389/fimmu.2021.668207 PMC8071949 33912195

[52] Gamrekelashvili J, Kapanadze T, Sablotny S, Ratiu C, Dastagir K, Lochner M, et al. Notch and TLR signaling coordinate monocyte cell fate and inflammation. eLife. 2020;9:e57007. 10.7554/eLife.57007 PMC7413669 32723480

[53] Dorneburg C, Goß AV, Fischer M, Roels F, Barth TF, Berthold F, et al. γ-Secretase inhibitor I inhibits neuroblastoma cells, with NOTCH and the proteasome among its targets. Oncotarget. 2016;7:62799–813. 10.18632/oncotarget.11715 PMC5325329 27588497

[54] Bray SJ. Notch signalling: a simple pathway becomes complex. Nat Rev Mol Cell Biol. 2006;7:678–89. 10.1038/nrm2009 16921404

[55] Golde TE, Koo EH, Felsenstein KM, Osborne BA, Miele L. γ-Secretase inhibitors and modulators. Biochim Biophys Acta. 2013;1828:2898–907. 10.1016/j.bbamem.2013.06.005 PMC3857966 23791707

[56] Fung E, Tang SM, Canner JP, Morishige K, Arboleda-Velasquez JF, Cardoso AA, et al. Delta-like 4 induces notch signaling in macrophages: implications for inflammation. Circulation. 2007;115:2948–56. 10.1161/CIRCULATIONAHA.106.675462 17533181

[57] Monsalve E, Ruiz-García A, Baladrón V, Ruiz-Hidalgo MJ, Sánchez-Solana B, Rivero S, et al. Notch1 upregulates LPS-induced macrophage activation by increasing NF-kappaB activity. Eur J Immunol. 2009;39:2556–70. 10.1002/eji.200838722 19662631

[58] Zeng Q, Jin C, Ao L, Cleveland JC Jr., Song R, Xu D, et al. Crosstalk between the Toll-like receptor 4 and Notch1 pathways augments the inflammatory response in the interstitial cells of stenotic human aortic valves. Circulation. 2012;126:S222–230. 10.1161/CIRCULATIONAHA.111.083675 PMC3506187 22965987

[59] Zeng Q, Song R, Ao L, Weyant MJ, Lee J, Xu D, et al. Notch1 promotes the pro-osteogenic response of human aortic valve interstitial cells via modulation of ERK1/2 and nuclear factor-kappaB activation. Arterioscler Thromb Vasc Biol. 2013;33:1580–90. 10.1161/ATVBAHA.112.300912 PMC3778193 23640488

[60] López-López S, Monsalve EM, Romero de Ávila MJ, González-Gómez J, Hernández de León N, Ruiz-Marcos F, et al. NOTCH3 signaling is essential for NF-κB activation in TLR-activated macrophages. Sci Rep. 2020;10:14839. 10.1038/s41598-020-71810-4 PMC7481794 32908186

[61] Ruan ZB, Fu XL, Li W, Ye J, Wang RZ, Zhu L. Effect of notch1,2,3 genes silicing on NF-κB signaling pathway of macrophages in patients with atherosclerosis. Biomed Pharmacother. 2016;84:666–73. 10.1016/j.biopha.2016.09.078 27697639

[62] López-López S, Romero de Ávila MJ, Hernández de León NC, Ruiz-Marcos F, Baladrón V, Nueda ML, et al. NOTCH4 exhibits anti-inflammatory activity in activated macrophages by interfering with interferon-γ and TLR4 signaling. Front Immunol. 2021;12:734966. 10.3389/fimmu.2021.734966 PMC8671160 34925319

[63] Khan H, Singh A, Thapa K, Garg N, Grewal AK, Singh TG. Therapeutic modulation of the phosphatidylinositol 3-kinases (PI3K) pathway in cerebral ischemic injury. Brain Res. 2021;147399. 10.1016/j.brainres.2021.147399 33662337

[64] Hoesel B, Schmid JA. The complexity of NF-kappaB signaling in inflammation and cancer. Mol Cancer. 2013;12:86. 10.1186/1476-4598-12-86 PMC3750319 23915189

[65] Li H, Chang C, Li X, Zhang R. The roles and activation of endocardial Notch signaling in heart regeneration. Cell Regen. 2021;10:3. 10.1186/s13619-020-00060-6 PMC7847831 33521843

[66] Marracino L, Fortini F, Bouhamida E, Camponogara F, Severi P, Mazzoni E, et al. Adding a “notch” to cardiovascular disease therapeutics: a MicroRNA-based approach. Front Cell Dev Biol. 2021;9:695114. 10.3389/fcell.2021.695114 PMC8435685 34527667

[67] Nistri S, Sassoli C, Bani D. Notch signaling in ischemic damage and fibrosis: evidence and clues from the heart. Front Pharmacol. 2017;8:187. 10.3389/fphar.2017.00187 PMC5381357 28424623

[68] Fortini F, Vieceli Dalla Sega F, Caliceti C, Aquila G, Pannella M, Pannuti A, et al. Estrogen receptor β-dependent Notch1 activation protects vascular endothelium against tumor necrosis factor α (TNFα)-induced apoptosis. J Biol Chem. 2017;292:18178–91. 10.1074/jbc.M117.790121 PMC5672041 28893903

[69] Ferrari R, Rizzo P. The Notch pathway: a novel target for myocardial remodelling therapy? Eur Heart J. 2014;35:2140–5. 10.1093/eurheartj/ehu244 24970336

[70] Rizzo P, Miele L, Ferrari R. The Notch pathway: a crossroad between the life and death of the endothelium. Eur Heart J. 2013;34:2504–9. 10.1093/eurheartj/ehs141 22645188

[71] Aquila G, Kostina A, Vieceli Dalla Sega F, Shlyakhto E, Kostareva A, Marracino L, et al. The Notch pathway: a novel therapeutic target for cardiovascular diseases? Expert Opinion on Therapeutic Targets. 2019;23:695–710. 10.1080/14728222.2019.1641198 31304807

[72] Kwon SM, Eguchi M, Wada M, Iwami Y, Hozumi K, Iwaguro H, et al. Specific Jagged-1 signal from bone marrow microenvironment is required for endothelial progenitor cell development for neovascularization. Circulation. 2008;118:157–65. 10.1161/CIRCULATIONAHA.107.754978 18591437

[73] Perrotta F, Perna A, Komici K, Nigro E, Mollica M, D’Agnano V, et al. The state of art of regenerative therapy in cardiovascular ischemic disease: biology, signaling pathways, and epigenetics of endothelial progenitor cells. Cells. 2020;9(8):1886. 10.3390/cells9081886 PMC7465688 32796767

[74] Giorgio V, Guo L, Bassot C, Petronilli V, Bernardi P. Calcium and regulation of the mitochondrial permeability transition. Cell Calcium. 2018;70:56–63. 10.1016/j.ceca.2017.05.004 28522037

[75] Pei H, Song X, Peng C, Tan Y, Li Y, Li X, et al. TNF-α inhibitor protects against myocardial ischemia/reperfusion injury via Notch1-mediated suppression of oxidative/nitrative stress. Free Radic Biol Med. 2015;82:114–21. 10.1016/j.freeradbiomed.2015.02.002 25680284

